# Factors influencing sexual behaviors among youths in Myanmar: the results from the Myanmar demographic and health survey 2015–16

**DOI:** 10.3389/frph.2025.1626266

**Published:** 2025-08-20

**Authors:** Yoon Shwe Yee Hlaing, Pallop Siewchaisakul, Sineenart Chautrakarn

**Affiliations:** Faculty of Public Health, Chiang Mai University, Chiang Mai, Thailand

**Keywords:** sexual behaviors, contraceptive use, early sexual initiation, paying for sex, youth, Myanmar demographic and health survey, reproductive health, influencing factors

## Abstract

**Background:**

Youths in Myanmar face heightened risks for adverse sexual health outcomes such as unintended pregnancies and sexually transmitted infections (STIs), including HIV/AIDS. Despite the increasing youth population in Myanmar, nationally representative data on factors influencing their sexual behaviors are limited. This study aimed to investigate the factors associated with sexual behaviors among youths in Myanmar.

**Methods:**

This study used data from the Myanmar Demographic Health Survey 2015–16 to analyze a sample of 4,645 youths aged 15–24. Descriptive statistics were used to investigate sociodemographic factors, HIV/STI and contraceptive knowledge, and sexual behaviors. Multivariable logistic regression was used to identify factors associated with sexual behaviors. Adjusted odds ratios (AOR) and 95% confidence intervals (CI) were presented. Statistical significance was determined at a *p*-value of <0.05.

**Results:**

Among sexually active youths (*n* = 1,366), 47.0% reported having their first sex at or before the age of 18, 54.0% were currently using contraception, and only 8.1% of males had paid for sex. Higher education levels, middle, richer, and richest wealth, as well as moderate and high contraception knowledge, were all protective against early sexual initiation. Females and currently married youths were more likely to use contraception, while regional disparities persisted, with youths from hilly, coastal, and plains regions less likely to use contraceptives than those from the delta and lowlands. Married males were less likely to pay for sex. Male youths with moderate or high contraception knowledge reported higher risk of paying for sex.

**Conclusion:**

According to this study, gender, education level, wealth status, marital status, region, and contraceptive knowledge all have a significant impact on sexual behaviors among Myanmar youths. Early sexual initiation and low contraceptive use are common, especially among young people with low education and socioeconomic status. Targeted, inclusive, and culturally sensitive sexual and reproductive health education and services are urgently needed to address knowledge gaps and promote safer behaviors among Myanmar's youth.

## Introduction

The World Health Organization (WHO) defines “adolescents” as individuals aged 10–19 years, “youth” as those aged 20–24 years, and “young people” as those aged 10–24 years (1). The definitions of these terms can vary depending on cultural norms, social expectations, and political considerations in different societies (2). Youths aged 15–24 account for 16% of the global population, or approximately 1.2 billion people (3). Of these young people, 87% live in developing countries (4), 62.4% in Asia, and 14.1% in Africa (5).

In Myanmar, around 16 million individuals fall within the 10–24 age group, making up 28% of the population. The youth population (aged 15–24) saw growth rates of 0.6% in 2001–02, 0.9% in 2004–05, and approximately 3.8% in 2007, showing a trend of increasing growth among young people (6).

Human Immunodeficiency Virus (HIV) and other Sexually Transmitted Infections (STIs) continue to be major public health concerns worldwide (7, 8). The last stage of HIV infection is acquired immunodeficiency syndrome (AIDS) (9). STIs are a broad category of infections spread primarily through sexual contact (8). As of 2023, approximately 39.9 million people worldwide were infected with HIV, and it remains a major global health issue with significant social and economic consequences. In 2023, approximately 1.3 million new HIV infections and 630,000 AIDS-related deaths were recorded (10). Every day, over one million people worldwide contract curable STIs (8).

Adolescents and youth account for a larger proportion of people living with HIV worldwide. In 2023 alone, there were 360,000 new HIV infections among people aged 15–24, with 140,000 adolescents aged 15–19 (11). According to UNAIDS, young people aged 15–24 will account for roughly one-quarter of all new HIV infections in the Asia-Pacific region in 2022 (12). Every year, approximately 333 million new cases of treatable sexually transmitted infections (STIs) are reported worldwide, with individuals aged 20–24 having the highest incidence, followed by those aged 15–19 (13).

In 2022, approximately 280,000 individuals in Myanmar were living with HIV. Around 6,400 people succumbed to HIV-related illnesses, and there were an estimated 11,000 new infections. Among youths aged 15–24, there were around 5,800 new HIV infections (12). According to World Bank data, the HIV prevalence in the 15–24 age group was 0.3% for both males and females in 2021 (14). Regarding STI data in Myanmar, the data is not well-documented. However, there are many STI cases among youths in clinical practice.

Sexual risk behaviors refer to actions that increase the likelihood of unplanned pregnancies or STIs, including HIV (15). These behaviors include premarital sex, early sexual initiation, and having unprotected sex with partners who might carry STIs, as well as not using contraception (16). A significant number of young people participate in sexual risk behaviors, leading to potential unintended health consequences (17).

Youths in Myanmar face significant health risks due to their sexual behaviors. Research indicates that in Myanmar, young people are participating in risky sexual behaviors, largely due to limited knowledge of sexual and reproductive health. For instance, 38.0% of adolescents are unaware that a woman can become pregnant from a single instance of sexual intercourse. Additionally, only 16.67% of individuals aged 15–24 possess accurate knowledge about preventing HIV transmission (18). While people in Myanmar have some awareness of condoms as a method to prevent sexually transmitted infections, including HIV/AIDS, their actual usage remains significantly low compared to other contraceptive methods. According to 2006 BSS data, condom use during high-risk sexual encounters was reported at 43.8% (19). A study on young people in Myanmar revealed that 11.9% had engaged in premarital sexual relationships. Still, consistent condom use among males was reported at only 36.6%, with 23.0% admitting to never using condoms (20). According to the United Nations Population Fund (UNFPA) of Myanmar, discussions about sexual and reproductive health are stigmatized, creating barriers for young people to access reliable information crucial for making informed decisions about their future (21).

There were some studies regarding sexual behaviors among youths. One study by focused on youths in Yangon (22), investigating factors related to the intention to prevent risky sexual behaviors. Similarly, a study focusing on underprivileged youth in Mandalay City (23). However, both studies utilized small sample sizes that represented specific townships, so their findings don't represent the entire country. That is why there is a need for more comprehensive nationwide research on sexual behavior among youths. Moreover, there is a lack of comprehensive research on sexual behaviors related to STIs and HIV/AIDS among youths in Myanmar.

Therefore, the objective of this study was to investigate the characteristics of youths, knowledge of STIs, including HIV/AIDS, knowledge of contraception, and sexual behaviors among youths in Myanmar. It utilized data from the Myanmar Demographic and Health Survey (MDHS) 2015–2016, a nationwide survey that includes various indicators related to sexual and reproductive health and HIV/AIDS. The MDHS was conducted in 2015–16, with the full report published in 2017. This study also sought to determine the association between these characteristics, knowledge of STIs including HIV/AIDS, knowledge of contraception, and sexual behavior among youths in Myanmar.

## Methods

### Data sources, study setting, and population

The data source for this study was the 2015–2016 Myanmar Demographic and Health Survey (MDHS). This survey is both the first of its kind and the most recent nationally representative population-based survey on Myanmar's demographics and health. Data collection occurred between December 7, 2015, and July 7, 2016. The MDHS employed a two-stage stratified sample design, allowing for the estimation of important indicators at the national, urban, rural, and state/region levels (24). The data set is from the DHS program. The study populations were Myanmar youths (aged 15–24). The inclusion criteria for this study included individuals aged 15–24 with knowledge of STIs, including HIV/AIDS, knowledge of contraception, and sexual behaviors. The exclusion criteria included individuals aged 15–24 with missing values. Finally, the sample size of this secondary data was 4,645.

### Study variables

The dependent variables in this study were: early age of first sex, current contraceptive use, and payment for sex among youths. For early age of first sex, responses indicating “first sexual experience occurred after age 18, such as 19, 20, 21, etc.”, were recoded as “no” In contrast, “first sexual experience at age 18 or younger such as 15, 16, 17,18” were recoded as “yes” for this study. For current contraceptive use, “not using” was recoded as “no”, while reported use of methods such as oral contraceptive pills, IUDs, injections, male condoms, and female sterilization was recoded as “yes”. For the variable paying for sex, the original binary responses (“yes” and “no”) were retained. The original survey questions related to these variables are as follows:

1. Early age of first sex - How old were you when you had sexual intercourse for the very first time?
2. Current contraceptive use - Are you currently doing something or using any method to delay or avoid getting pregnant? Which method are you using?
3. Paying for sex - Have you ever paid anyone in exchange for having sexual intercourse?

The independent variables consisted of the age, gender, marital status, region, residence (urban/rural), education level, occupation, wealth Index, currently using tobacco products, and mass media exposure, heard about other STIs, level of knowledge of HIV/AIDS, level of knowledge of contraception. Level of knowledge of HIV/AIDS and level of knowledge of contraception were classified into 3 levels: Low (less than 60%), Moderate (between 60% and 79%), and High (between 80% and 100%), based on Bloom's cut-off point (25–28).

### Data analysis

STATA 15.1 was used for data analysis. According to the latest DHS guideline (DHS-8), we use weighted data using the functions v005/1,000,000 and mv005/1,000,000 and STATA survey setting (svyset) command (29). The descriptive statistics were used for characteristics of the participants, knowledge of STIs including HIV/AIDS, knowledge of contraception, and sexual behaviors (early age of first sex, current contraceptive use, and paying for sex) to find frequency and percentage. The association of independent variables (characteristics, knowledge of STIs including HIV/AIDS, knowledge of contraception) and dependent variables (sexual behaviors) were examined by using multivariable logistic regression analyses. Results were reported using adjusted odds ratios (AOR) with 95% confidence intervals (CI). A significance level of *p*-value <0.05 was set for statistical significance.

## Results

### Characteristics of youths

Table 1 presents the characteristics of the study sample. Of the 4,645 youths, the distribution was nearly equal between the 15–19 (49.2%) and 20–24 (50.8%) age groups. The majority were females (72.1%). In terms of marital status, most participants (72.9%) reported being single. Two-thirds of youths were from rural areas (66.7%), with a notable representation from delta and lowland region (45.7%). Most participants had secondary-level education (62.9%) and were employed (63.9%). Wealth index of the participants was almost evenly distributed. Only 8.2% smoked cigarettes, 0.2% chewed tobacco, and overall, 8.4% used tobacco products. Regarding exposure to media, 22.4% of the participants read newspapers or magazines, 30.5% of the participants listened to the radio; meanwhile, an extraordinary proportion of 69.7% of the youths watched television. Overall, 77.9% of youths had exposure to at least one media in the past 12 months.

**Table 1:** Characteristics of the youths (15–24 years) in Myanmar (*n* = 4,645).

Variables	*n*	Weighted (%)	Unweighted (%)
Age group			
15–19	2,294	49.2	49.4
20–24	2,351	50.8	50.6
Gender			
Male	1,321	27.9	28.4
Female	3,324	72.1	71.6
Marital status			
Single	3,379	72.9	72.7
Currently married	1,183	25.4	25.5
Widowed/Divorced/separated	83	1.7	1.8
Region			
Delta and lowland	1,668	45.7	35.9
Hilly	1,097	13.5	23.6
Coastal	575	8.0	12.4
Plains	1,305	32.8	28.1
Residence (urban/rural)			
Urban	1,496	33.3	32.2
Rural	3,149	66.7	67.8
Education level			
No education	155	3.7	3.4
Primary	1,086	24.7	23.4
Secondary	2,984	62.9	64.2
Higher	420	8.7	9.0
Employment status			
Unemployment	1,752	36.1	37.7
Employment	2,893	63.9	62.3
Wealth index			
Poorest	684	14.9	14.7
Poorer	836	17.3	18.0
Middle	1,068	22.3	23.0
Richer	1,068	22.6	23.0
Richest	989	22.9	21.3
Exposure to media - reading a newspaper or magazine			
No	3,620	77.6	77.9
Yes	1,025	22.4	22.1
Exposure to media - listening to radio			
No	3,226	69.5	69.5
Yes	1,419	30.5	30.5
Exposure to media - watching television			
No	1,501	30.3	32.3
Yes	3,144	69.7	67.7
Mass media exposure			
No access	1,115	22.1	24.0
Access to at least one media	3,530	77.9	76.0
Currently smoking cigarettes			
No	4,230	91.8	91.1
Yes	415	8.2	8.9
Currently chewing tobacco			
No	4,634	99.8	99.8
Yes	11	0.2	0.2
Currently using tobacco products			
No	4,222	91.6	90.9
Yes	423	8.4	9.1

### Knowledge of STIs, including HIV/AIDS

Table 2 demonstrates knowledge of SITs including HIV/ AIDS among youths. Regarding overall HIV knowledge, 38.3% had high knowledge, 32.0% had moderate knowledge, and 29.7% had low knowledge indicating knowledge level varied. In HIV/AIDS prevention, 64.8% of youths knew that they could reduce transmission of HIV by using condom. Misconceptions regarding HIV/AIDS were predominant, for example, 39.4% of youths responded that HIV could not be transmitted from mosquito bites, showing a significant misconception. Similarly, 41.5% of youths responded that HIV could be transmitted by sharing food with an HIV/AIDS infected person. Moderate level of knowledge of mother-to-child transmission was detected and 73.3% of participants correctly responded that HIV can be transmitted during pregnancy, 59.2% during delivery, and 67.9% through breastfeeding. Awareness of HIV testing was moderate, with 66.0% knowing where to get tested. Furthermore, 76.6% denied the belief that HIV might be transmitted through witchcraft. Participants had low level of awareness of STIs, only 20.4% of youths heard about other STIs.

**Table 2:** Knowledge of HIV/AIDS and STIs among youths (15–24 years) in Myanmar (*n* = 4,645).

Variables	*n*	Weighted (%)	Unweighted (%)
Ever heard of HIV/ AIDS			
No	0	0.0	0.0
Yes	4,645	100.0	100.0
Reduce chances of getting HIV by using condom			
Not correct	1,596	35.2	34.4
Correct	3,049	64.8	65.6
Reduce chance of getting HIV by having just one uninfected sex partner			
Not correct	1,232	25.9	26.5
Correct	3,413	74.1	73.5
Can get HIV from mosquito bites			
Not correct	2,791	60.6	60.1
Correct	1,854	39.4	39.9
Can get HIV by sharing food			
Not correct	1,928	41.5	41.5
Correct	2,717	58.5	58.5
Can a healthy-looking person have HIV			
Not correct	1,632	34.8	35.1
Correct	3,013	65.2	64.9
HIV can be transmitted during pregnancy			
Not correct	1,155	26.7	24.9
Correct	3,490	73.3	75.1
HIV can be transmitted during delivery			
Not correct	1,818	40.8	39.1
Correct	2,827	59.2	60.9
HIV can be transmitted by breastfeeding			
Not correct	1,456	32.1	31.3
Correct	3,189	67.9	68.7
Know a place to be tested for HIV			
No	1,600	34.0	34.4
Yes	3,045	66.0	65.6
Can get HIV by witchcraft or supernatural means			
Not correct	1,193	23.4	25.7
Correct	3,452	76.6	74.3
Level of knowledge of HIV/ AIDS			
Low (0–6)	1,392	29.7	30.0
Moderate (7–8)	1,475	32.0	31.7
High (9–11)	1,778	38.3	38.3
Heard about other STIs			
No	3,641	79.6	78.4
Yes	1,004	20.4	21.6

### Knowledge of contraception

Table 3 shows the knowledge of contraception among youths in Myanmar. The majority of youths were aware of at least one contraceptive method (97.0%). Exposure to family planning (FP) messages was relatively low. Only 11.8% reported hearing FP statements on the radio in the previous 12 months, while 24.1% had seen them on television and 20.9% had read about them in newspapers or magazines. Overall knowledge of contraception, the majority of youths scored low (63.7%), 19.9% scored moderate, and just 16.4% scored high.

**Table 3:** Knowledge of contraception among youths (15–24 years) in Myanmar (*n* = 4,645).

Variables	*n*	Weighted (%)	Unweighted (%)
Knowledge of any contraceptive method			
Don't know any method	142	3.0	3.1
Know any method (Modern or Traditional)	4,503	97.0	96.9
Heard family planning (FP) on radio last months			
No	4,117	88.2	88.6
Yes	528	11.8	11.4
Heard FP on TV last months			
No	3,617	75.9	77.9
Yes	1,028	24.1	22.1
Heard FP on newspaper or magazine last months			
No	3,714	79.1	80.0
Yes	931	20.9	20.0
Level of knowledge of contraception			
Low (0–1)	3,050	63.7	65.7
Moderate (2)	888	19.9	19.1
High (3–4)	707	16.4	15.2

### Sexual behaviors

As shown in Table 4, out of the 1,366 sexually active youths, 47.0% had their first sexual experience at or before the age of 18. In terms of current contraceptive use, 54% used a method, with injectable contraception (31.1%) being the most popular, followed by oral contraceptives (20.1%). However, 47% did not use any contraceptive method. Out of the 319 sexually active male youths, only 8.1% paid for sex.

**Table 4:** Sexual behaviors among sexually active youths (15–24 years) in Myanmar (*n* = 1,366).

Variables	*n*	Weighted (%)	Unweighted (%)
Early age of first sex			
Not early age of first sex (>18 years)	706	53.0	51.7
Early age of first sex (≤18 years)	660	47.0	48.3
Current contraceptive methods			
Not using	698	46.0	51.1
OC pills	243	20.1	17.8
IUD	13	0.9	0.9
Injections	380	31.1	27.8
Male condom	4	0.2	0.3
Female sterilization	2	0.3	0.1
Periodic abstinence	4	0.2	0.3
Withdrawal	9	0.4	0.7
Implants/Norplant TM	8	0.5	0.6
Lactational amenorrhea (lam)	1	0.1	0.1
Other modern method	4	0.2	0.3
Current contraceptive use			
Not using	698	46.0	51.1
Using	668	54.0	48.9
Paying for sex^a^ (*n* = 329)			
No	297	91.9	90.3
Yes	32	8.1	9.7

^a^ Male only because the variable is only present for males in the original data set.

### Factors influencing sexual behaviors

Table 5 presents univariable and multivariable logistic regression models of factors influencing early age of first sex, current contraceptive use, and paying for sex. Accordingly, females were more likely than males to experience early sexual initiation (AOR = 2.50, 95% CI: 1.51–4.12, *p* < 0.001). Participants who had high education level had significantly lower odds (AOR = 0.62, 95% CI: 0.43–0.89, *p* = 0.010) of reporting early age of first sex than those with low education level. Similarly, youths who had middle, richer, and richest wealth index had significantly lower odds (AOR = 0.37, 95% CI: 0.23–0.60, *p* < 0.001, AOR = 0.40, 95% CI: 0.25–0,65, *p* < 0.001, AOR = 0.32, 95% CI: 0.18–0.58, *p* < 0.001, respectively) of early age of first sex than poorest one. Current tobacco users had significantly higher odds of initiating sex early compared to non-users (AOR = 1.85, 95% CI: 1.01–3.39, *p* = 0.046). Participants who had moderate and high level of knowledge of contraception had lower odds of having early age of first sex (AOR = 0.57, 95% CI: 0.37–0.88, *p* = 0.010, AOR = 0.50, 95% CI: 0.30–0.83, *p* = 0.008, respectively) than those with low level one. Regarding contraceptive use, females were more likely than males to use contraceptives (AOR = 1.78, 95% CI: 1.16–2.74, *p* = 0.009). Currently married youths had increased odds of contraceptive use (AOR = 2.78, 95% CI: 1.43–5.40, *p* = 0.003) while formerly married youths had decreased odds of contraceptive use (AOR = 0.11, 95% CI: 0.03–0.35, *p* < 0.001). The odds of using contraceptives were significantly lower among those who resided in hilly, coastal, and plain regions (AOR = 0.34, 95% CI: 0.22–0.50, *p* < 0.001, and AOR = 0.49, 95% CI: 0.33–0.73, *p* < 0.001, AOR = 0.61, 95% CI: 0.43–0.86, *p* = 0.004 respectively) than those in delta and lowland region. For paying for sex, married males had decreased odds of engaging in paying for sex compared to single males (AOR = 0.02, 95% CI: 0.01–0.09, *p* < 0.001). Male youths who had moderate or high level of knowledge of contraception had higher odds of paying for sex than low-level ones (AOR = 7.69, 95% CI: 1.95–30.24, *p* = 0.004).

**Table 5:** Univariable and multivariable logistic regression analysis of factors influencing sexual behaviors among sexually active youths in Myanmar.

Variables	Early age of first sex		Current contraceptive use		Paying for sex	
	Adjusted OR (95% CI)	*P*-value	Adjusted OR (95% CI)	*P*-value	Adjusted OR (95% CI)	*P*-value
Age group						
15–19	ref		ref		ref	
20–24	0.05 (0.03–0.08)	<.0001*	1.21 (0.85–1.71)	0.291	2.58 (0.63–10.60)	0.187
Gender						
Male	ref		ref		ref	
Female	2.50 (1.51–4.12)	<.0001*	1.78 (1.16–2.74)	0.009*	1^a^	
Marital status						
Single	ref		ref		ref	
Currently married	0.61 (0.32–1.18)	0.140	2.78 (1.43–5.40)	0.003*	0.02 (0.01–0.09)*^,b^	<0.001*^,b^
Formerly married (Widowed, Divorced, No longer living together/separated)	1.00 (0.41–2.45)	1.000	0.11 (0.03–0.35)	<0.001*		
Region						
Delta and lowland	ref		ref		ref	
Hilly	1.35 (0.83–2.18)	0.226	0.34 (0.22–0.50)	<0.001*	1.31 (0.27–6.44)	0.741
Coastal	0.95 (0.59–1.53)	0.829	0.49 (0.33–0.73)	<0.001*	0.62 (0.01–3.91)	0.606
Plains	1.02 (0.71–1.48)	0.909	0.61 (0.43–0.86)	0.004*	0.30 (0.08–1.06)	0.061
Residence (urban/rural)						
Urban	ref		ref		ref	
Rural	0.72 (0.49–1.05)	0.091	0.72 (0.48–1.08)	0.115	0.43 (0.13–1.45)	0.174
Education level						
Low education level	ref		ref		ref	
High education level	0.62 (0.43–0.89)	0.010*	1.07 (0.77–1.50)	0.676	0.70 (0.20–2.41)	0.569
Employment status						
Unemployment	ref		ref		ref	
Employment	0.84 (0.61–1.15)	0.270	1.15 (0.86–1.55)	0.35	0.91 (0.08–9.95)	0.94
Wealth index						
Poorest	ref		ref		ref	
Poorer	0.63 (0.39–1.02)	0.059	1.19 (0.73–1.92)	0.483	1.08 (0.13–9.20)	0.943
Middle	0.37 (0.23–0.60)	<0.001*	1.20 (0.77–1.88)	0.426	0.88 (0.13–5.81)	0.893
Richer	0.40 (0.25–0.65)	<0.001*	0.99 (0.59–1.67)	0.981	1.16 (0.25–5.29)	0.852
Richest	0.32 (0.18–0.58)	<0.001*	0.77 (0.42–1.42)	0.407	0.09 (0.01–0.86)	0.037*
Mass media exposure						
No access	ref		ref		ref	
Access to at least one media	1.32 (0.95–1.83)	0.094	1.08 (0.80–1.45)	0.609	1.01 (0.31–3.26)	0.985
Currently using tobacco products						
No	ref		ref		ref	
Yes	1.85 (1.01–3.39)	0.046*	1.05 (0.62–1.80)	0.848	0.82 (0.22–3.02)	0.768
Heard about other STIs						
No	ref		ref		ref	
Yes	0.92 (0.65–1.31)	0.657	1.37 (0.93–1.99)	0.107	0.49 (0.15–1.61)	0.236
Level of knowledge of HIV/ AIDS						
Low (0–6)	ref		ref		ref	
Moderate (7–8)	0.81 (0.55–1.19)	0.280	1.17 (0.80–1.71)	0.413	2.98 (0.82–10.81)	0.096
High (9–11)	1.15 (0.76–1.74)	0.509	1.48 (1.01–2.18)	0.045*	1.26 (0.31–5.21)	0.745
Level of knowledge of contraception						
Low (0–1)	ref		ref		ref	
Moderate (2)	0.57 (0.37–0.88)	0.010*	1.13 (0.78–1.64)	0.525	7.69 (1.95–30.24)^c^	0.004*^,c^
High (3–4)	0.50 (0.30–0.83)	0.008*	0.86 (0.56–1.31)	0.469		

^a^ Omitted because only include male participants.

^b^ Recode Currently married, Formerly married into Ever married.

^c^ Recode Moderate, High into Moderate and high.

* Significant at *p*-value <0.05.

## Discussion

This study is the first study that explores sexual behavior among Myanmar's youths nationwide. The overall prevalence of early age of first sex among youths was 47.0%, indicating that a sizable proportion of youths engage in sexual activities before reaching the age of consent. This finding is very similar to that of Debre Markos University students in North West Ethiopia (44.6%) (30). This finding is higher than those reported among teenage students in Kiryandongo District, Uganda (20.0%) (31), adolescents and youths in Benin (31.7%) (32), and college students in Southwest Ethiopia (17.9%) (33). This significant percentage of youth emphasizes the importance of promoting safe sexual practices among young people.

The overall prevalence of current contraceptive use among youths was found to be 54.0%. This finding is higher than reported among unmarried youths in Yangon, Myanmar (44%) (34) and among adolescents in the Tamale Metropolis, Ghana (29.4%) (35). On the other hand, it is lower than those of young people in Southern Ethiopia (67.6%) (36) and of Adolescents in Techiman Municipality, Ghana (65%) (37). This finding indicates that only half of the youths used contraception, increasing the risk of unintended pregnancies and STIs, including HIV.

The prevalence of transactional sexual relationships among male youths was only 8.1%. This figure is lower than those reported for unmarried male youths in India (17.24%) and Cambodia (82.5%) (38, 39). The relatively low reported rate of paying for sex may be due to survey participants' social desirability bias, or it may reflect the true prevalence, necessitating additional research using a variety of approaches.

The findings revealed that female youths were more likely than male youths to engage in early sexual initiation. This is consistent with previous research conducted in Ethiopia (40) and Uganda (31). One possible reason for this is that in many cultures, female sexuality is more strictly judged and controlled by society than male sexuality. This social pressure can shape how females behave, possibly leading to earlier sexual experiences due to power imbalances or social expectations. High levels of education and economic status (as measured by the wealth index) were protective factors against early sexual initiation. These findings are consistent with a previous study in Uganda (41), where socioeconomic empowerment and education protect against an early sexual debut. Furthermore, youths with moderate and high contraception knowledge had lower odds of having their first sex at a young age, demonstrating the protective effect of reproductive health awareness programs.

In terms of contraceptive use, the study discovered that female youths were more likely to use contraception than males, contradicting findings from other studies, such as Nepal (42) and Brazil (43). This could be due to a greater use of female-centered methods such as injectables and pills, as well as increased involvement in family planning after marriage in Myanmar. Current marital status was a strong predictor, with currently married people being more likely to use contraception. This finding is consistent with that of Ghana (35), indicating that contraceptive use increases during marriage due to family planning considerations. Formerly married youths, on the other hand, were less likely to use contraception, which could be due to a lack of need or the social stigma associated with using contraception after marriage. Contraception was less commonly used in hilly, coastal, and plain regions than in the delta and lowland regions. This could be due to inequalities in outreach programs, healthcare provider availability, and access to health infrastructure. Geographic disparities in healthcare delivery must be addressed through targeted service delivery and youth-friendly outreach in underserved areas.

Furthermore, the analysis revealed that male youths who reported ever marrying were significantly less likely to report paying for sex than singles, which is consistent with previous research conducted in Cambodia (38) and Sub-Saharan African countries (44), implying that social or moral obligations exist within marital contexts. However, one intriguing finding was that male youths with moderate to high levels of contraceptive knowledge were more likely to engage in transactional sex. This pattern has also been observed among male youths in Nigeria (45), where higher contraceptive knowledge is associated with increased transactional sex. This result may seem counterintuitive, as it is generally expected that increased awareness of contraception would lead to safer and more responsible sexual practices. However, several cultural and psychosocial factors may contribute to this result. In some contexts, greater contraceptive knowledge may arise not from formal sex education or health initiatives, but from peer influence or exposure to sexualized media, which can simultaneously promote and normalize risky sexual behaviors like paying for sex. Furthermore, young men with higher contraceptive knowledge may develop a false sense of confidence, believing that their knowledge of contraception can sufficiently shield them from adverse outcomes. This overconfidence could reduce their perception of risk and increase the likelihood of engaging in unsafe sexual practices. Cultural norms regarding masculinity and sexual dominance may further reinforce this condition, particularly in societies where transactional sex is viewed as a marker of male virility or social status. It is also important to recognize that having access to contraception and sexual health information does not automatically lead to responsible or ethical decision-making. This gap between knowledge and actual behavior highlights the need for comprehensive sexual education that goes beyond technical understanding, incorporating discussions around values, consent, gender norms, and emotional intelligence to shape behavior more effectively.

### Limitations

The study's key strength is the use of a large, nationally representative MDHS dataset, allowing for generalizable conclusions. The use of both univariable and multivariable logistic regression strengthens the validity of the associations found.

However, this study has some limitations that must be acknowledged. The cross-sectional design of the MDHS data prevents causal interpretations. Responses to sexual behaviors are subject to recall and social desirability bias. The dataset did not include variables such as peer pressure, alcohol use, exposure to pornography, cultural norms, and familial dynamics, which may also influence youth sexual behaviors.

Additionally, this study may be affected by measurement errors resulting from the way variables were recoded. For example, converting early age of first sex into a categorical variable (“yes early” vs. “no early”) and classifying knowledge levels using Bloom's cut-off may have oversimplified complex behaviors and knowledge gradients. Although such transformations can be necessary for analysis, they may also introduce misclassification bias. Furthermore, variable like paying for sex was only available for male respondents, which limited the scope of analysis and reduced generalizability across genders. The lack of equivalent data for female participants constrains the interpretation of sexual behaviors among the broader youth population, particularly in cultural contexts where female transactional sex may be underreported or differently defined. Future research should adopt more inclusive data collection and nuanced measurement methods to achieve a more comprehensive and equitable representation of all youth subpopulations.

### Policy and practice implications

The findings from the MDHS data study have significant policy and practice implications. Education, both formal and informal, remains an important factor in delaying sexual initiation and promoting safe sexual behaviors. Interventions must target out-of-school and underserved areas to bridge knowledge gaps. Efforts to improve contraception access and use should prioritize reducing regional disparities. Mobile health clinics, community-based distribution programs, and culturally sensitive outreach campaigns may help to increase contraceptive use, especially in hilly, coastal, and plain areas where use is low. Understanding the motivations and conditions for transactional sex in male adolescents is critical. Programs should aim to improve not only knowledge but also attitudes and perceptions of masculinity, sexual risk-taking, and relationships.

## Conclusion

According to this study, gender, education level, wealth status, marital status, region, and contraceptive knowledge all have a significant impact on sexual behaviors among Myanmar youths. Early sexual initiation and low contraceptive use are common, especially among young people with low education and socioeconomic status. These findings highlight the critical need for targeted, comprehensive sexual and reproductive health education and services that are inclusive, youth-friendly, and culturally sensitive to better equip Myanmar's youth with the knowledge and resources they require to make safe and informed decisions.

## Data Availability

The raw data supporting the conclusions of this article will be made available by the authors, without undue reservation.
